# Editorial: Rethinking Juvenile Recidivism: Towards a More Holistic View of Success

**DOI:** 10.3389/fpsyg.2024.1416703

**Published:** 2024-06-12

**Authors:** Tom D. Kennedy, Kendell L. Coker, Derek T. Hess, David Shapiro

**Affiliations:** ^1^Nova Southeastern University, Fort Lauderdale, FL, United States; ^2^Department of Psychology, Connecticut College, New London, CT, United States; ^3^State of Iowa Department of Health and Human Services, Des Moines, IA, United States

**Keywords:** juvenile justice, juvenile offender, rehabilitation, recidivism, juvenile justice programs, risk factors

Recidivism has long been the primary outcome measure for juveniles involved with the justice system. As a “fundamental concept of criminal justice” (NIJ, 2008), recidivism generally refers to a person's continuation or relapse into illegal behavior. It is an indicator commonly used to evaluate how well the juvenile justice system, including its services and supports, has rehabilitated youth and reduced the likelihood of them engaging in future delinquent or criminal acts.

This primary focus on re-offending seems reasonable when viewed from a more correctional or justice system perspective. Recidivism data, for instance, inform correctional and delinquency-based policy, particularly regarding interventions and sanctions. If approaches do not ultimately reduce a youth's delinquent behavior, then why would we adopt them?

After decades of using this simplistic and dichotomous (recidivate or not) approach to assess and understand delinquent and criminal behavior, we have learned that it is no longer sufficient to rely on recidivism as the primary measure of success for youth involved with the justice system for offending. We are not suggesting that recidivism is immaterial. Rather, it alone fails to provide clarity concerning the complex and nuanced challenges faced by youth and families, their individualized and dynamic needs, and the drivers of success.

As a prelude to this special edition, this editorial is designed to highlight reasons against continuing to use a primarily recidivism-based approach to help youth desist delinquent acts. This special edition is focused on concepts like (a) the lack of an operational definition of recidivism, (b) the disproportionate impact of a recidivism focus on some minoritized communities, (c) recidivism's inability to account for deep-rooted systemic or structural forces that contribute to recidivism (Caudill and Trulson, 2023), and (d) how its deficit-based framework runs contrary to “rehabilitative” ideals for youth involved with the justice system. Overall, we hope that this special edition acts as a call to action for scholars, researchers, practitioners, administrators, and policy influencers to achieve a more holistic approach for youth involved with the justice system.

## Operational Definitions of Recidivism

There is no uniform method for defining or measuring recidivism, which makes it difficult to compare recidivism rates across programs and jurisdictions. Juvenile justice studies generally define recidivism as a new delinquency referral (e.g., offense and rearrest), a new adjudication, or a new sentence (e.g., incarceration), and its measurement may vary based on the length of time between discharge from a program or release from incarceration (Robertson et al., 2020). For instance, some studies might explore whether a juvenile recidivated within 1 year, while other studies use a 2-year time frame or longer.

Further compounding these disparities in measurement is the fact that some programs serve youth with higher risk factors for recidivism than others. Rearrest is a crude but simple approach because it is easy to capture these data from official police records. However, this approach is also highly problematic. First, an arrest does not mean the person committed the offense, and therefore, using this operational definition for recidivism arguably carries the implied underpinnings of guilt presumption in a system that presumes innocence until proven guilty. The United States Sentencing Commission (2022) noted that using rearrests to measure recidivism results in higher recidivism rates than reconviction or reincarceration, which is, in part, because the evidentiary standard for an arrest (probable cause) is lower than that which is required for a conviction (beyond a reasonable doubt). In addition, Black/Brown youth are generally more likely than their white peers to be arrested or have police contact irrespective of prior justice involvement, thereby contributing to biased estimates of recidivism.

The use of a new adjudication or sentence is a way to mitigate this concern. However, there is uncertainty regarding when the case will be adjudicated, which creates challenges in measuring recidivism in a timely manner. Access to zealous legal representation impacts court outcomes as does bias in forensic evaluations of Black/Brown youth (Kennedy et al., 2023), both of which increase racial disparities in criminal and civil cases among minority youth. In addition, the racism/discrimination that exists at every stage of the juvenile justice system further contributes to disparities in adjudicative decisions and biased recidivism estimates (Evangelist et al., 2017; Childs et al., 2024).

## Overemphasis on Recidivism Is a Problem

Most juveniles are not incarcerated for their offenses, and, in part, due to the de-institutionalization of status offenders, they are diverted or sentenced to probation and/or community sanctions (Kennedy et al., 2020; Puzzanchera et al., 2022). Researchers have begun to account for the unique risk factors for recidivism depending on the placement for youth involved with the legal system. Despite the relative improvement, this does not adequately address the problem that lies at the core of over-reliance on recidivism to measure the effectiveness of a juvenile program. For example, it is not uncommon for recidivism as the primary outcome measure to influence programmatic funding decisions (NCSL, 2023). The reality is that over-reliance on recidivism does not accurately capture the juvenile's current functioning, wellness, or progress due to their involvement in programs, which is supposed to be the intended focus of the rehabilitative framework of the juvenile justice system.

Instead of focusing on recidivism as the primary measure of success, it is important for programs and systems for youth involved with the legal system for offending to stress the need and value of helping young people develop into productive and responsible citizens, not just reducing rates of delinquent or criminal behavior. There are certainly more nuanced, encouraging, and strength-based changes experienced by youth while attending rehabilitative programs that often are not focused on or measured and may not show up until later in life (Cavanagh, 2022). In other words, it is critical to emphasize reductions in both criminogenic and non-criminogenic needs and the building of strengths rather than just reductions in ultimate recidivism rates. This finding is in line with the full risk-need-responsivity (RNR) model (Ward et al., 2007; Andrews et al., 2011; Bonta and Andrews, 2017). For beyond the core RNR principles, the model also includes overarching principles such as respect for the person and normative context and prioritizing crime prevention efforts. It is important to strike the right balance between interest in crime prevention (e.g., reduction in recidivism rates) with due regard for the holistic human nature of youth and families, including the incorporation of developmental perspectives. An optimally balanced approach is poised to protect adolescents from overcriminalization while also holding them accountable for their actions.

Recidivism rates are affected by several factors that are beyond the control of offender programs for youth involved with the legal system for offending, such as the socioeconomic status of the youth's family, the level of crime and negative peer influences in the youth's neighborhood, and the youth's behavioral health. In addition, the typical definitions of recidivism fail to capture the complete picture of the nuanced systemic forces (Caudill and Trulson, 2023) that impact a juvenile's risk level (e.g., epigenetics, discrimination, and a lack of access to support and services). In addressing these crucial concerns, program developers should evaluate existing efforts and explore innovative interventions.

Furthermore, recidivism rates do not measure the full range of benefits provided by programs for youth involved with the legal system for offending. For example, programs for youth involved with the legal system for offending may help young people develop new skills (e.g., emotional regulation, distress tolerance, communication, and aggression replacement), improve their bonds with their families and support system, and form positive relationships and effective communication with others. Using recidivism rates as the main outcome variable or marker of success may discourage programs for youth involved with the legal system from refining their approach to behavior management and expanding their intervention models and resources. Programs for youth involved with the legal system that are focused solely on reducing recidivism rates may be less likely to attempt new and novel approaches that could be more effective in helping young people change their risky behavior and desist from delinquent or criminal behavior. In the same manner that objectives are stepwise progressions to goals in treatment plans, skill development essential to reducing recidivism may be missed if we only focus on the end goal.

## Redefining Outcomes Toward a More Comprehensive Approach

This editorial highlights a few of the primary considerations for moving away from a recidivism-centric model to assess success with youth involved with the justice system. The subsequent articles in this special edition provide more nuanced considerations for jurisdictions and programs to consider.

Overall, we need to focus on a wider range of outcomes that more holistically assess “whether” and “how” a youth and their support system are improving. For example, prosocial behaviors like whether young people are completing their education, finding gainful and prosocial employment, promoting positive youth development, and building positive relationships with their families and communities may elucidate how social determinants of health can reduce the risk of offending (Cavanagh, 2022). Given that youth involved with the justice system are often referred to system-based interventions (e.g., evidence-based family therapy), we have an opportunity to identify and assess more creative, holistic ways to measure improved functioning within their family/kinship networks (e.g., communication and familial conflict) while also removing barriers to access and increase engagement (Amani et al., 2018). This review highlights the importance of addressing the underlying stressors faced by youth involved with the justice system and their families, which often drive their system involvement. It also stresses the importance of helping youth and their support systems develop healthier coping mechanisms to mitigate the risk factors associated with the unitary construct of recidivism (National Academies of Sciences, Engineering, and Medicine, 2022). Concentrating on broad-ranging outcomes across a longer timeframe may help ensure that some funding for programs for youth involved with the legal system is allocated to pioneering, strength-based, holistic programs. It is this detail, nuance, and context that we believe is essential for ultimately achieving the shared goal of reducing recidivism for youth who are involved with the justice system.

## Author contributions

TK: Conceptualization, Writing – original draft, Writing – review & editing. KC: Conceptualization, Writing – original draft, Writing – review & editing. DH: Conceptualization, Writing – original draft, Writing – review & editing. DS: Writing – review & editing.
